# Virucidal effects of eucalyptus essential oil on porcine reproductive and respiratory syndrome virus

**DOI:** 10.3389/fmicb.2024.1443295

**Published:** 2024-08-20

**Authors:** Tianbao Chen, Baoling Liu, Dingyu Liu, Qin Luo, Yani Wang, Xiaohu Wang, Dongsheng He, Rujian Cai

**Affiliations:** ^1^Key Laboratory of Livestock Disease Prevention of Guangdong Province, Scientific Observation and Experiment Station of Veterinary Drugs and Diagnostic Techniques of Guangdong Province of Ministry of Agriculture and Rural Affairs, Institute of Animal Health, Guangdong Academy of Agricultural Sciences, Guangzhou, China; ^2^College of Veterinary Medicine, South China Agricultural University, Guangzhou, China; ^3^College of Veterinary Medicine, Huazhong Agricultural University, Wuhan, China

**Keywords:** porcine reproductive and respiratory syndrome virus (PRRSV), Eucalyptus essential oil (EEO), antiviral, virucidal effects, PRRS (porcine reproductive and respiratory syndrome virus)

## Abstract

Currently, the efficacy of vaccination for preventing and controlling PRRSV is insufficient. Therefore, there is an urgent need for novel effective preventive strategies. This study aimed to investigate the antiviral effect of Eucalyptus essential oil (EEO) against PRRSV *in vitro*. Marc-145 cells were infected with PRRSV (rJXA1-R), and the toxicity of EEO in the cells was measured using the Cell Counting Kit-8 method. Additionally, the antiviral effect of EEO on PRRSV-infected cells was assessed using three treatment methods: drug administration post-PRRSV inoculation (post-treatment), drug administration before PRRSV inoculation (pre-treatment), and simultaneous drug administration and PRRSV inoculation (co-treatment). The EEO could not inhibit virus adsorption and/or replication since post-treatment and pre-treatment did not prevent viral infectivity. However, EEO exerted a significant virucidal effect on PRRSV. When PRRSV-infected cells were treated with 0.0156, 0.0312, and 0.0625% EEO, the cell survival rates were 55.37, 118.96, and 121.67%, respectively, and the titer of progeny virions decreased from 5.77 Log_10_TCID_50_ to 5.21 Log_10_TCID_50_, 0.55 Log_10_TCID_50_, and less than 0.167 Log_10_TCID_50_, respectively (where TCID_50_ is the 50% tissue culture infected dose). The fluorescence intensity of the PRRSV N protein significantly decreased in the indirect immunofluorescence assay. When cells were co-treated with EEO (0.0625%) and PRRSV (1000 TCID_50_) for 15 min, the viral particles were inactivated, and PRRSV (1000 TCID_50_) particles loss infectivity when the co-treatment time reached 60 min. In a word, EEO has no obvious therapeutic effect on PRRSV infection, but it can effectively inactivate virus particles and make them lose the ability to infect cells. These findings provide insights for the development and use of EEO to treat PRRS.

## 1 Introduction

Porcine reproductive and respiratory syndrome (PRRS) is a highly virulent infectious disease caused by the porcine reproductive and respiratory syndrome virus (PRRSV). It is clinically characterized by reproductive disorders in sows and respiratory diseases in pigs of all ages (Zimmerman et al., 1997). PRRSV is an enveloped single-stranded positive-sense RNA virus. It belongs to the genus *Arterivirus* [along with equine arteritis virus (EAV), simian hemorrhagic fever virus (SHFV), and lactate dehydrogenase-elevating virus (LDV)] and the family *Arteriviridae* (Snijder et al., 2013). It can be classified into European and North American genotypes. The PRRSV genome length is approximately 15 kb, contains a 5′ untranslated region (5’UTR) with a cap structure and a 3’UTR with a polyA tail, which includes 10 open reading frames (ORFs) (Montaner-Tarbes et al., 2019).

Vaccine immunization is the primary method to prevent and control PRRSV infection. However, the cross-protective effect of the vaccine is insufficient owing to the high variability of PRRSV (Liu et al., 2018). With the recent advancements in biotechnology, various antiviral strategies have been developed, such as CD163 receptor knockout in pigs, host microRNA, antisense RNA, nanobodies, immune stimulants, and neutralizing targets (Du et al., 2017). However, these technologies are in the research stage and there are no effective commercial antiviral drugs to combat PRRSV infection. Natural plants and their extracts are important resources for drug development, and many natural plant extracts inhibit PRRSV replication (Du et al., 2017; Ge et al., 2018; Yu et al., 2019; Long et al., 2022; Yu et al., 2022). Essential oils (EOs) are a type of natural plant extract. They have been widely studied owing to their antibacterial-, antifungal-, and antioxidant properties (Valdivieso-Ugarte et al., 2019). Essential oils from various aromatic plants and herbs have antiviral effects (Ma and Yao, 2020). Pellegrini et al. (2023) demonstrated the significant virucidal effects of lemon EO (LEO) using a cultivatable norovirus (NoV) surrogate, feline calicivirus (FCV) (Pellegrini et al., 2023). *Thymus vulgaris* EO (TEO) has antiviral and virucidal effects against feline infectious peritonitis virus (FIPV) in CrFK cells (Catella et al., 2021). Ginger EO (GEO) has antiviral activity against caprine alphaherpesvirus 1 (CpHV-1) *in vitro*; it is effective as a virucide against cell-free virions, with a 100% inactivation rate against CpHV-1 (Camero et al., 2019).

Eucalyptus essential oil (EEO) is generally obtained by steam distillation or hydrodistillation of leaves and its main chemical constituent is monoterpenoids, as well as a small amount of sesquiterpenoids (Čmiková et al., 2023). It is commonly used to treat respiratory diseases like colds, bronchitis, and pharyngitis. It exhibits antibacterial, anti-inflammatory-, antioxidant-, antiviral-, and antiparasitic activities (Dhakad et al., 2018). Furthermore, it has an inhibitory effect on respiratory viral infections, such as herpes simplex viruses (HSVs), influenza viruses (IVs), SARS-CoV-2 (COVID-19), mumps virus (MuV), coxsackievirus (CV), and rotaviruses (RVs) (Mieres-Castro et al., 2021). In this study, we aimed to investigate the antiviral activity of EEO against PRRSV infection *in vitro* and determine whether EEO could inactivate PRRSV, thus reducing PRRSV infection in cells. The findings of this study highlight the potential of EEO as a natural option for PRRSV control strategies.

## 2 Materials and methods

### 2.1 Cells and virus strains

Marc-145 cells were cultured in Dulbecco’s modified Eagle’s medium (DMEM; Gibco, Waltham, MA, United States) supplemented with penicillin/streptomycin 100 IU/mL and 10% fetal bovine serum (FBS; Gibco, Waltham, MA, United States) in a cell culture incubator (37°C, 5% CO_2_). Highly pathogenic type 2 PRRSV (PRRSV-2) strain JXA1-R (GenBank ID: MT163314) was purchased from Guangdong Winsun Bio Pharmaceutical Company (Guangzhou, China). PRRSV rJXA1-R was amplified and titrated into Marc-145 cells, resulting in a virus titer of 10^6.7^ TCID_50_.

### 2.2 Antibodies

Mouse monoclonal antibodies against the PRRSV N protein were purchased from Guangzhou Qianxun Biotechnology (Guangzhou, China). Goat anti-mouse secondary antibodies conjugated with Alexa Fluor® 488 were purchased from Cell Signaling Technology (MA, United States).

### 2.3 Composition analysis of EEO

Eucalyptus essential oil was purchased from Nanjing Jiulonghui Fragrance Company (Nanjing, China). The composition of EEO was validated using gas chromatography with mass spectrometry (GC/MS), which was performed by Guangzhou Weiping Technology Service (Guangzhou, China).

Gas chromatography conditions: inlet temperature, 280°C; temperament interface temperature, 280°C; carrier gas flow rate, 1.5 mL/min; sample size, 1 μL; shunt ratio, 10:1. The heating procedure included an initial temperature of 50°C, heating at 5°C/min to 100°C for 5 min, heating at 4°C/min to 140°C for 5 min, heating at 4°C/min to 180°C for 5 min, heating at 5°C/min to 250°C for 5 min, and heating at 10°C/min to 290°C for 10 min.

Mass spectrometry conditions: ion source temperature, 230°C; quadrupole temperature, 150°C; Electrospray ionization (EI), 70 eV; and full scanning, 35–550 da.

### 2.4 Cytotoxicity assay

A Cell Counting Kit-8 assay (CCK-8; Beyotime, Shanghai, China) was used to examine the cytotoxicity of EEO in Marc-145 cells. First, EEO was dissolved in dimethyl sulfoxide (DMSO) which dilution is 0.5%, then EEO was added to DMEM for dilutions. Marc-145 cells were inoculated into 96-well plates, and different concentrations of EEO (0.0039, 0.0078, 0.0156, 0.0312, 0.0625, 0.125, 0.25, and 0.5%) were added when the cells had grown to 80–90% confluence. Untreated cells and cells treated with equivalent dilutions of DMSO (without EEO) served as the control and blank controls, respectively. After incubation for 72 h incubation, 10 μL CCK-8 was added to each well and incubated for another 2 h. Finally, the fluorescence value was determined using the Multiskan-Spectrum (Thermo Fisher Scientific, MA, United States) at an absorbance of 450 nm. The relative viability of the cells was calculated using the formula: cell survival rate (%) = [OD (sample) − OD (blank) / OD (control) − OD (blank)] × 100%.

### 2.5 Antiviral activity assay

**Figure 1:**
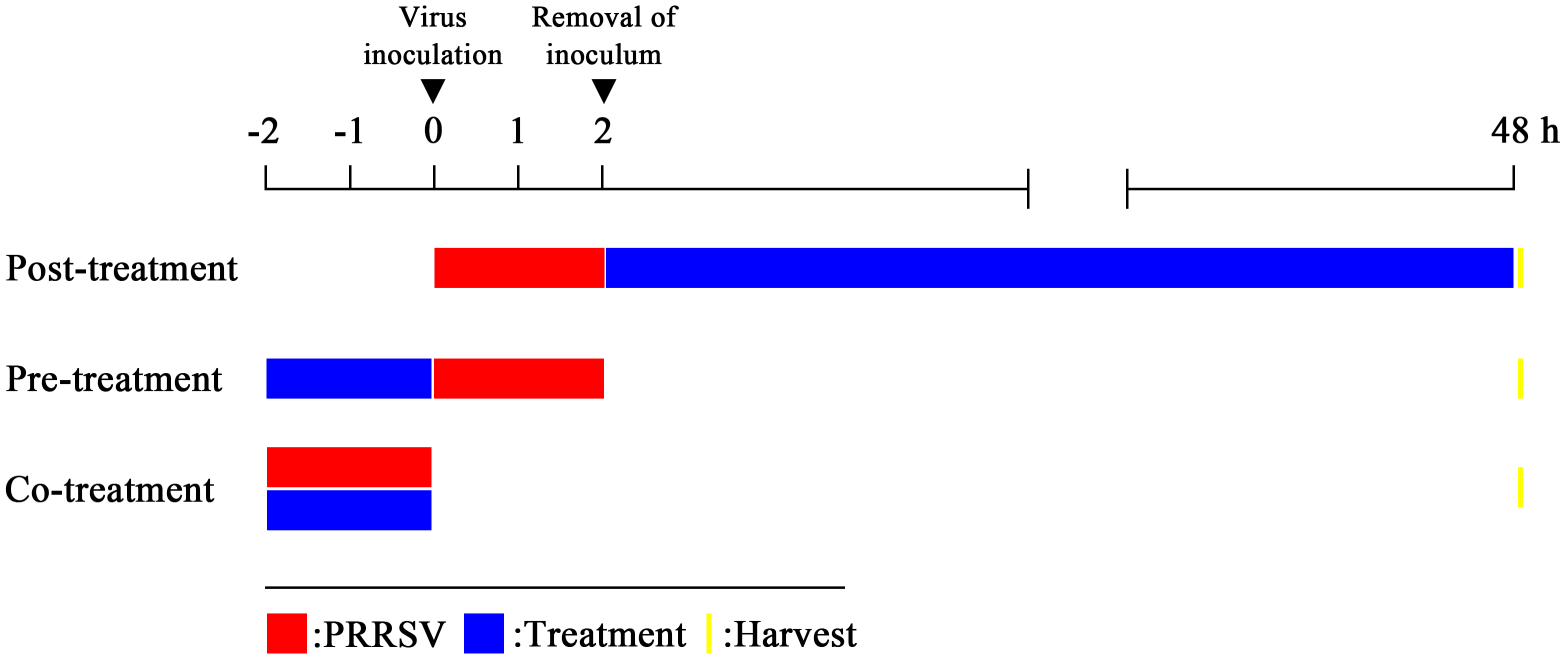
Three treatment modes. Post-treatment: virus-infected cells are treated with an antiviral substance; pre-treatment: cells are treated with an antiviral substance before virus inoculation to the cells; co-treatment: virus mixed with an antiviral substance and then the mixture is inoculated to the cells.

To identify the mechanism of PRRSV inhibition by EEO, three different protocols (A, B, and C) were used (Figure 1). Monolayers of Marc-145 cells grown in 24-well plates were used. Four experimental groups were established: 1. the drug treatment groups: EEO treatment (0.0625, 0.0312, and 0.0156%); 2. the positive control group: Ribavirin (Rib) treatment; 3. the virus control group: DMEM treatment; 4. the cell control group: DMEM treatment. Dimethyl sulfoxide (at a final concentration of 0.5%) was added to each group. All experiments were performed in triplicate.

#### 2.5.1 Protocol a: post-treatment with EEO

Marc-145 cells were inoculated with 1000 μL PRRSV (1000 TCID_50_) for 2 h at 37°C. After three washes with PBS, different concentrations of EEO or Rib were added to the cells. In the virus and the cell control groups, maintenance medium containing 0.5% DMSO was added. After 48 h, the viral titer was determined and indirect immunofluorescence analysis was performed.

#### 2.5.2 Protocol B: pre-treatment with EEO

Marc-145 cells were pre-treated with different concentrations of EEO or Rib for 2 h at 37°C. After three washes with PBS, 1,000 μL PRRSV (1,000 TCID_50_) was added and incubated for 2 h. The inoculum was removed, the cell monolayers were washed three times with PBS, and a maintenance medium containing 0.5% DMSO was added. After 48 h, the viral titer was determined and indirect immunofluorescence analysis was performed.

#### 2.5.3 Protocol C: co-treatment with EEO

Two-fold dilutions of EEO or Rib were mixed with a 2x TCID_50_ PRRSV diluent (after mixing, the drug and virus concentrations were halved) for 2 h. Subsequently, the mixture was inoculated into the cells and cultured for 2 h. After the cell monolayers were washed three times with PBS, and a maintenance medium containing 0.5% DMSO was added. After 48 h, the viral titer was determined and indirect immunofluorescence analyses were performed.

### 2.6 Viral titration (TCID_50_)

Cells and supernatants were collected and subjected to three freeze–thaw cycles at −80 and 4°C, respectively, to ensure maximum release of virions from cells. Ten-fold serial dilutions of PRRSV were inoculated into Marc-145 cells in 96-well plates. After 2 h of incubation at 37°C, the medium was replaced with fresh DMEM containing 2% FBS. Viral titers were calculated after 72 h using endpoint dilution analysis. The Karber method was used to calculate the 50% tissue culture infected dose (TCID_50_).

### 2.7 Indirect immunofluorescence assay

Indirect immunofluorescence assays were performed as previously described (Zhang et al., 2018), with a few modifications to observe PRRSV-infected and uninfected cells. Briefly, cells were fixed with 4% paraformaldehyde for 15 min. After permeabilization with 0.25% Triton X-100 for 15 min at room temperature (RT), the cells were blocked with 2% bovine serum albumin for 60 min. The cells were incubated with a mouse monoclonal antibody against the PRRSV N-protein (1:1,000 dilution) at 4°C overnight. Subsequently, cells were incubated with anti-mouse secondary antibodies conjugated with Alexa Fluor^®^488 (green) (1:1,000 dilution) for 2 h. Nuclei were stained using DAPI (Beyotime, Shanghai, China) (blue), and the cells were examined under a fluorescence microscope (Leica, Wetzlar, Germany). The blue and green fluorescent spots were counted to obtain the total and PRRSV-infected cell numbers, respectively, in each IFA image.

### 2.8 Cell survival rates analysis

Marc-145 cells were seeded into 96-well plates. The steps of the analysis of the antiviral effect were as follows: first, the drug treatment, virus control, and cell control groups were prepared as described in section 2.5. When the cytopathic effect induced by the virus of cells in the virus control group reached 80–90%, 10 μL CCK-8 was added to each well for 2 h. Fluorescence was detected at 450 nm. Cell viability calculation formula: cell survival rate (%) = [OD (drug) − OD (virus)/OD (blank) − OD (virus)] × 100% (Cheng et al., 2013).

### 2.9 Statistical analysis

All experiments were performed at least three times. Statistical analyses were performed using GraphPad Prism software (version 8.0; GraphPad, CA, United States) and significant differences between groups were determined using one-way or two-way analysis of variance. *p*-values <0.05 were considered statistically significant and are denoted by “*” in the figures.

## 3 Results

### 3.1 Composition of EEO

**Figure 2:**
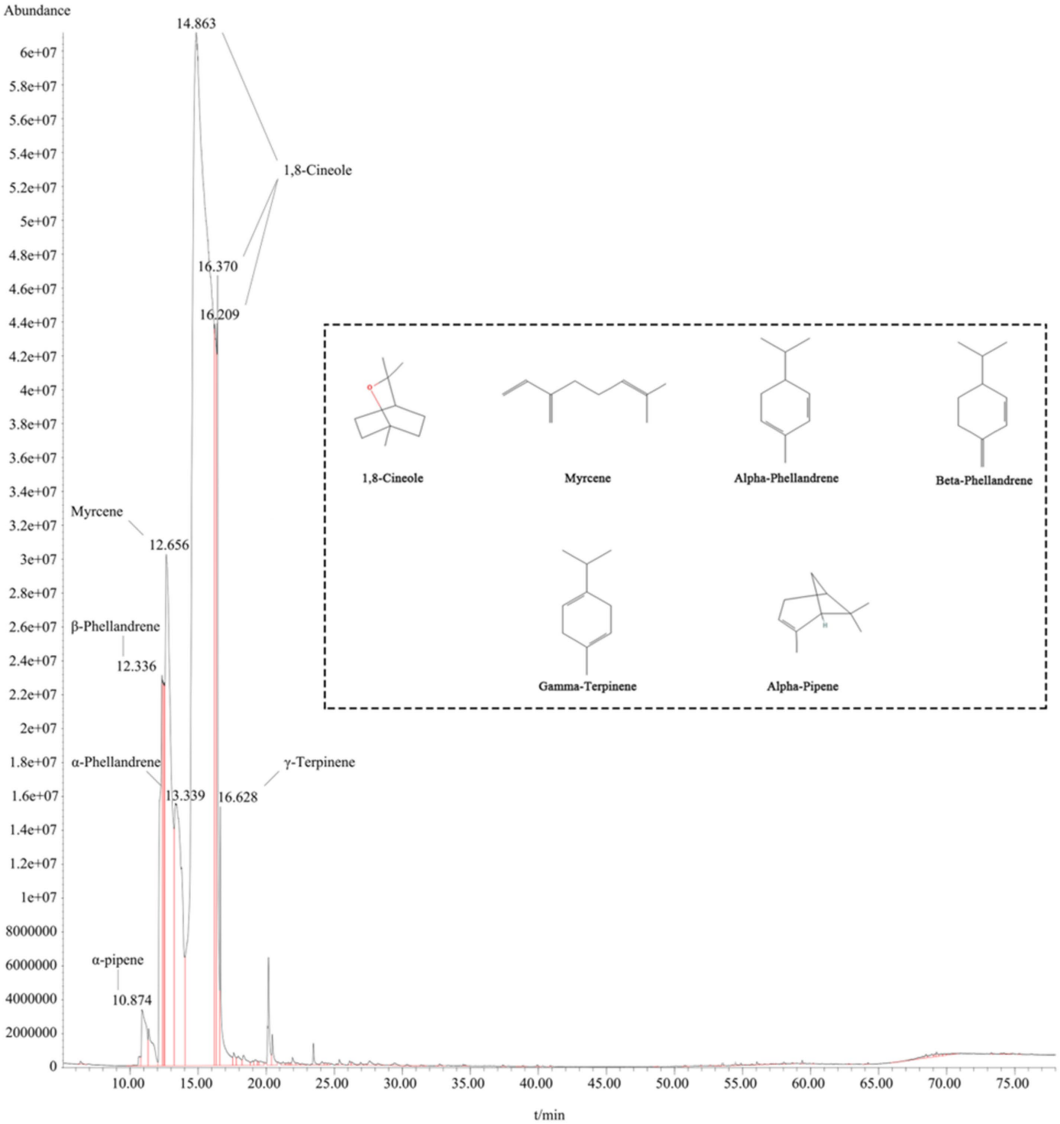
Post-treatment with EEO. The inset displays the molecular structure formula of the top six compounds based on peak area ratio.

The chemical composition of the EEO was characterized using GC/MS analysis. The major components of EEO were identified by comparing its mass spectral data with the mass spectral data stored in the National Institute of Standards and Technology (NIST) database (Figure 2). A total of 92 components were identified in EEO, with 15 components comprising 94.91% of the total content (Table 1). Under fixed chromatographic conditions, the peak area of the sample was directly proportional to its concentration. The top six compounds based on peak area were 1,8-cineole (71.4146%), myrcene (10.8169%), alpha-phellandrene (6.852%), beta-phellandrene (3.263%), gamma-terpinene (1.2081%), and alpha-pinene (0.8935%).

**Table 1:** Chemical composition of EEO.

PK	RT	Area (%)	Library/ID	CAS	SI/MS
1	10.675	0.0751	Alpha-Thujene	002867-05-2	95
2	10.8747	**0.8935**	**Alpha-Pinene**	000080-56-8	96
3	12.3378	**3.263**	**Beta-Phellandrene**	000555-10-2	96
4	12.655	**10.8169**	**Myrcene**	000123-35-3	97
5	13.3366	**6.852**	**Alpha-Phellandrene**	000099-83-2	91
6	14.8642	**63.1154**	**1,8-Cineole**	000470-82-6	99
7	16.2098	**4.4614**	**1,8-Cineole**	000470-82-6	92
8	16.3684	**3.8378**	**1,8-Cineole**	000470-82-6	93
9	16.6269	**1.2081**	**Gamma-Terpinene**	000099-85-4	96
10	17.614	0.106	Alpha-Terpinolene	000586-62-9	95
11	18.3426	0.1477	P-Cymene	000099-87-6	94
12	21.9325	0.0569	4-Isopropyl-2-Cyclohexenone	000500-02-7	86
13	34.4828	0.0055	Alpha-Humulene	006753-98-6	95
14	34.6238	0.0051	Beta-Santalene	000511-59-1	86
15	37.3912	0.0062	Beta-Cadinene	000523-47-7	95
16	53.5196	0.0412	M-Camphorene	020016-73-3	99
17	54.4656	0.0188	P-Camphorene	020016-72-2	99

Bold font, Top six compounds in EEO; PK, Compounds are listed in the order of elution from the column; Rt, Retention time of the compounds. Area (%) is the percentage of one or more peak areas in the chromatogram to the total peak area. SI/MS, Similarity Index/Mass Spectrum (NIST. Database).

### 3.2 Cytotoxicity

**Figure 3:**
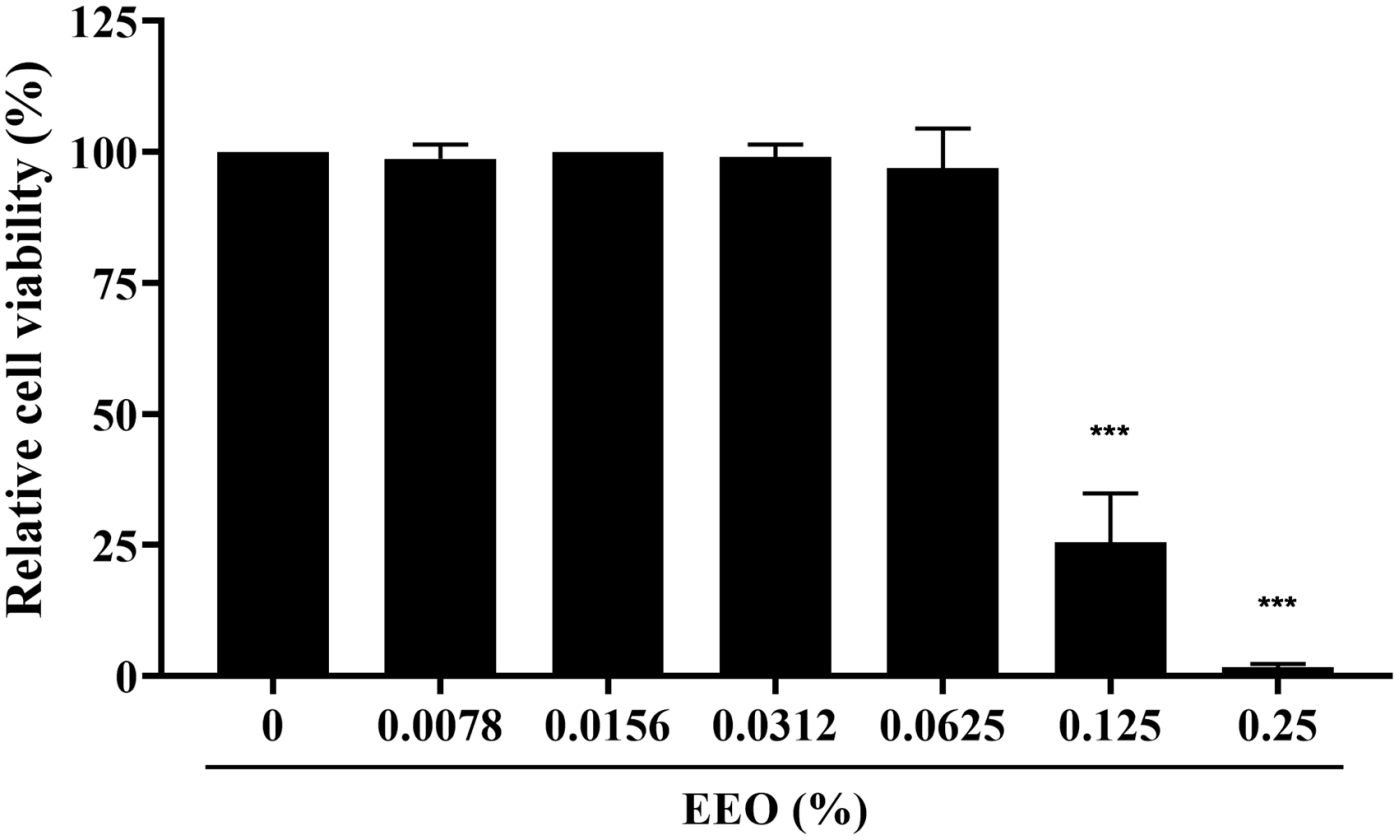
Cytotoxicity of EEO in Marc-145 cells.

To determine the cytotoxicity of EEO, we used the CCK-8 assay with various EEO concentrations in Marc-145 cells, which were used for *in vitro* PRRSV infection. As shown in Figure 3, EEO did not impair Marc-145 cell viability at concentrations <0.0625%. However, EEO significantly affected Marc-145 cell viability at concentrations ranging from 0.125 to 0.25%. The 50% cytotoxic concentration (CC50) of EEO in Marc-145 cells was 0.1054%.

### 3.3 Antiviral activity

#### 3.3.1 Protocol a: post-treatment with EEO

**Figure 4:**
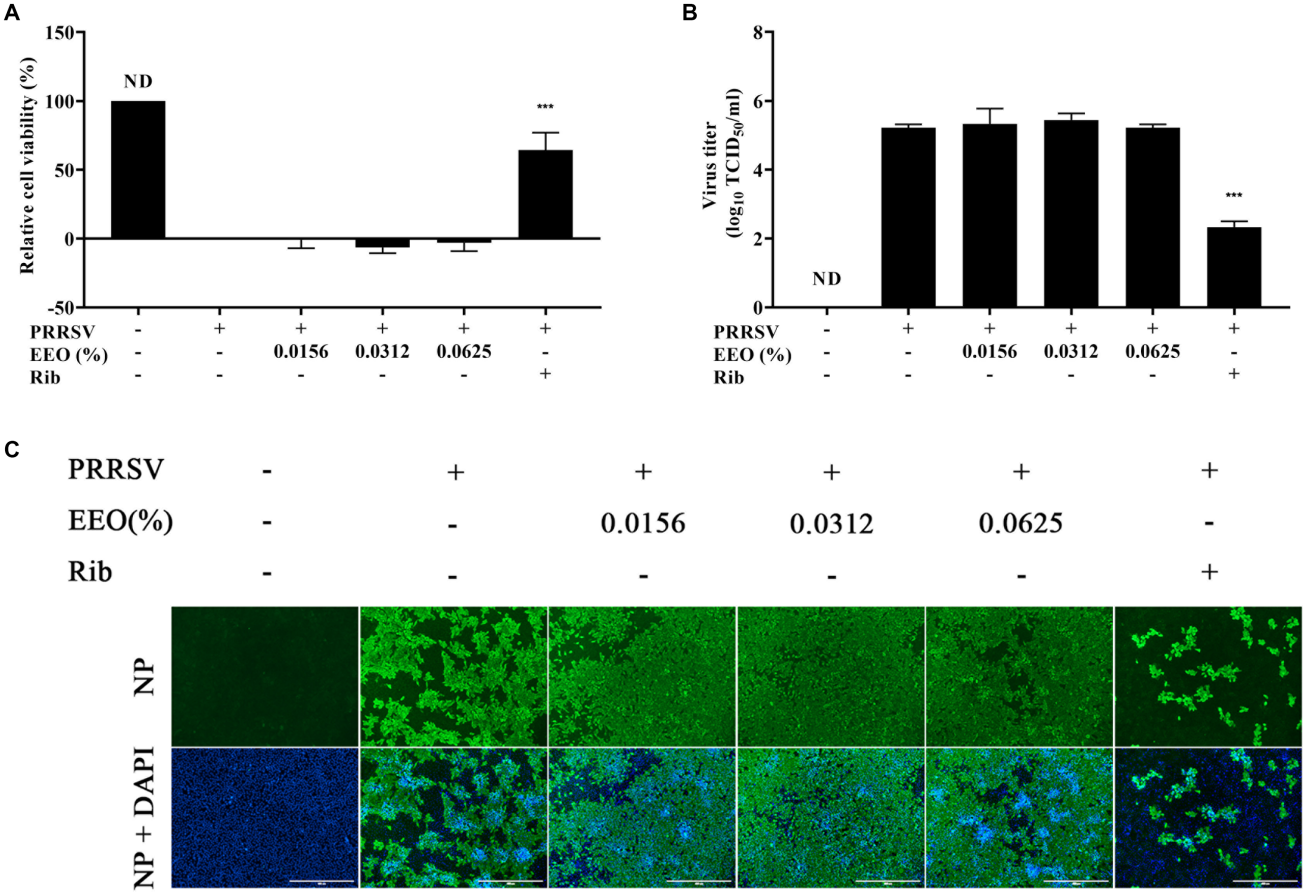
Post-treatment with EEO. **(A)** Cell survival rate, **(B)** viral titration, and **(C)** indirect immunofluorescence assays were performed in the treated and untreated groups at specific times after infection. Ribavirin was used for the drug control group.

Subsequently, we explored whether EEO treatment administered after PRRSV infection of Marc-145 cells had an inhibitory effect on PRRSV proliferation. A CCK8 assay, viral titer assay, and indirect IFA were conducted. The cell survival rate of Rib against PRRSV measured using the CCK8 method was 64.30%, which was significantly higher than that of the viral control group (Figure 4A). The viral titer of the Rib group in the viral titer assay was 2.33 Log_10_TCID_50_, which was 2.89 Log_10_TCID_50_ lower than that of the virus control group (5.22 Log_10_TCID_50_) and indicated a significant reduction (Figure 4B). There was no significant difference in PRRSV N protein fluorescence intensity between the EEO and virus control groups under all drug treatment protocols, whilst that in the Rib group was significantly lower than that in the virus control group according to IFA (Figure 4C). Additionally, there was no significant difference in PRRSV N protein fluorescence between the different EEO concentrations in the control groups. In summary, post-treatment of EEO did not effectively inhibit PRRSV infection.

#### 3.3.2 Protocol B: pre-treatment with EEO

**Figure 5:**
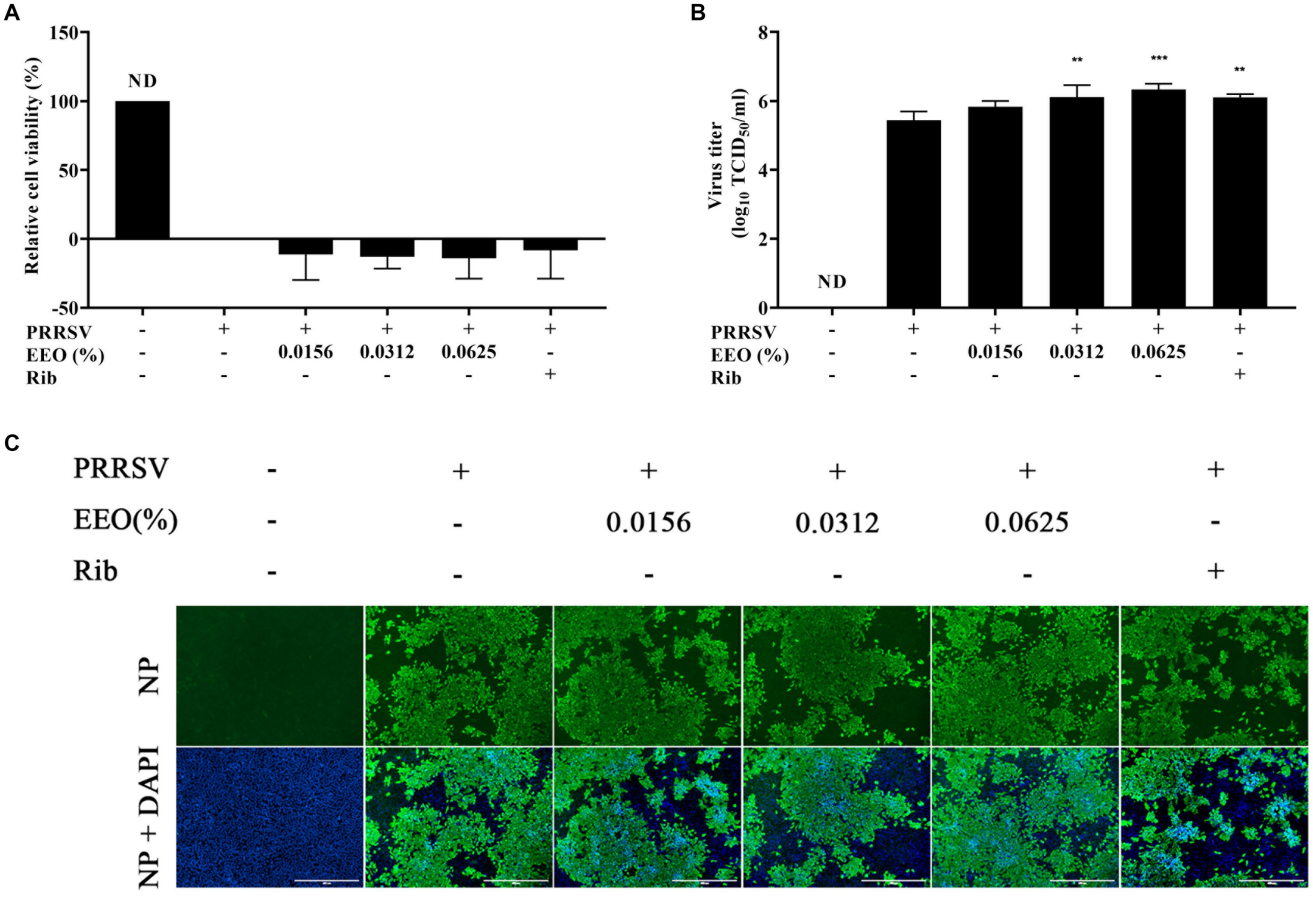
Pre-treatment with EEO. Drug administration before PRRSV inoculation. **(A)** Cell survival rate, **(B)** viral titration, and **(C)** indirect immunofluorescence assays were performed in the treated and untreated groups at specific times after infection. Ribavirin was used in the drug control group.

Cells were pretreated with different concentrations of EEO or Rib before PRRSV infection to determine whether EEO pretreatment could alter the susceptibility of Marc-145 cells to PRRSV. The cell survival rate in the EEO and Rib treatment groups was lower than that of the virus control group (Figure 5A). The virus titration assay and IFA confirmed that EEO pre-treatment did not alter the susceptibility of the cells to PRRSV (Figures 5B,C).

#### 3.3.3 Protocol C: co-treatment with EEO

**Figure 6:**
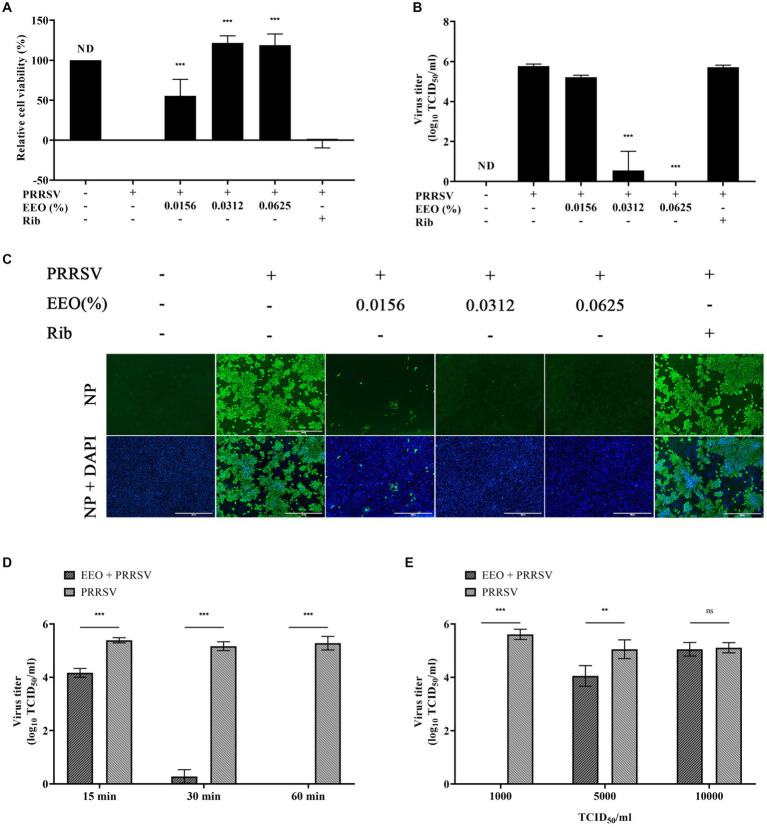
Co-treatment with EEO. Simultaneous drug administration and PRRSV inoculation. **(A)** Cell survival rate assay, **(B)** viral titration assay, and **(C)** IFA of the treated and untreated groups were performed at specific times after infection. Ribavirin was used for the drug control group. **(D)** Co-treatment with EEO and PRRSV for different durations or **(E)** Co-treatment of EEO with different TCID_50_ PRRSV values.

To explore whether EEO could inactivate PRRSV, a 2x concentration of EEO and 2000 TCID_50_ of PRRSV were added to Marc-145 cells. The cell survival rate of 0.0156% EEO concentration was 55.37%, while those of 0.0312 and 0.0625% EEO were 118.96 and 121.67%, respectively (Figure 6A). The cell survival rates of EEO at all three concentrations against PRRSV were significantly higher than those in the virus control group. When the EEO concentration was 0.0156%, the viral titer of PRRSV progeny was 5.22 Log_10_TCID_50_, which was 0.55 Log_10_TCID_50_ lower than that of the viral control group (5.77 Log_10_TCID_50_). In contrast, the viral titers of PRRSV progeny were 0.55 Log_10_TCID_50_ and less than 0.167 Log_10_TCID_50_ at EEO concentrations of 0.0312 and 0.0625%, respectively, which were significantly lower than those of the virus control group (Figure 6B). Additionally, IFA showed that the fluorescence intensity of the PRRSV N protein was significantly lower than that of the virus control group after treatment of PRRSV-infected cells with different concentrations of EEO (Figure 6C). These results indicate that EEO interacts with PRRSV virions in some way and affects their infectivity in cells.

To further explore the inactivation effect of EEO on PRRSV virions, the maximum safe concentration of EEO was used to study the effects of different treatment times (15, 30, and 60 min) and viral titers (1,000 TCID_50_, 5,000 TCID_50_, and 10,000 TCID_50_). Cells were infected with 0.0625% EEO and 1,000 TCID_50_ PRRSV for different durations. The treatment groups with different viral titers were treated with 0.0625% EEO and different TCID_50_ of PRRSV for 60 min. The cytotoxic effect was analyzed using a viral titer assay. After 15 min of interaction between EEO and PRRSV, the viral titer of the virions in the EEO group (4.16 Log_10_TCID_50_) significantly decreased by 1.22 Log_10_TCID_50_ compared with that in the viral control group (5.38 Log_10_TCID_50_) (Figure 6D). The inactivation effect of EEO significantly increased with an increase in treatment time. The viral titer of the PRRSV progeny decreased to less than 0.167 Log_10_TCID_50_ when EEO was co-treated with 1000 TCID_50_ PRRSV for 60 min. The viral titer in the EEO group (4.05 Log_10_TCID_50_) significantly decreased by 1 Log_10_TCID_50_ compared with that in the viral control group (5.05 Log_10_TCID_50_) after the interaction of EEO with 5000 TCID_50_ PRRSV for 60 min (Figure 6E). The interaction of EEO and 1000 TCID_50_ PRRSV for 15 min effectively inactivate virions, and 1000 TCID_50_ PRRSV virions were eradicated when the treatment time reached 60 min.

## 4 Discussion

The emergence of PRRSV has had a serious impact on the global pig industry. Pigs infected with PRRSV have high mortality rates. Currently, PRRS is mainly prevented and controlled by vaccinations, including attenuated and inactivated vaccines. However, these vaccines cannot effectively prevent and control PRRSV infections owing to safety and efficacy limitations. Therefore, it is necessary to identify new strategies for combating PRRSV infections. Many studies show that plant EOs have good antiviral properties (Ma and Yao, 2020). *Eucalyptus* is a tree genus belonging to the Myrtaceae family and many studies demonstrate that compounds extracted from eucalyptus have antibacterial, antiviral, and anti-inflammatory properties (Salehi et al., 2019).

In this study, we demonstrated the antiviral activity of EEO against PRRSV *in vitro*. Three drug concentrations that were below the cytotoxic threshold were selected for testing. Ribavirin was selected as the positive drug control because it inhibits PRRSV replication in Marc-145 cells (Khatun et al., 2015). In pre-and post-drug treatment experiments, we found that EEO did not significantly increase the cell survival rate or reduce the viral titer and PRRSV N protein fluorescence intensity. In previous studies, on drug treatment before viral infection, natural plant compounds reduced the adsorption and invasion of PRRSV-susceptible cells by altering the expression of receptors, such as CD163 and CD151 (Ge et al., 2018). However, we found that EEO did not block viral infection in our study, indicating that EEO does not inhibit infection by altering the expression of PRRSV-susceptible cell receptors. From the perspective of drug treatment after viral infection, EEO cannot inhibit post-viral infection processes, such as replication. Our experiments confirmed that ribavirin inhibits PRRSV replication, supporting the findings of the previous study.

Furthermore, we assessed the virucidal activity of EEO, which was effective against cell-free virions. Treatment with EEO and PRRSV for 1 h significantly increased the survival rate of infected cells and decreased the viral titer and fluorescence intensity of the PRRSV N protein. When the concentration of EEO decreased, PRRSV death decreased in a dose-dependent manner. The virus particles were significantly inactivated when EEO interacted with PRRSV (1000 TCID_50_) at the maximum safe drug concentration (0.0625%) for 30 min. After 60 min, the viruses were completely inactivated. The PRRSV titer decreased when EEO interacted with PRRSV (1000 TCID_50_) at the maximum drug safety concentration (0.0625%) for 15 min. The PRRSV titer significantly decreased with an increase in treatment time to 60 min (The decrease was 22.65% at 15 min, 94.65% at 30 min, and the virus was completely inactivated at 60 min). This indicates that the exposure time is an important factor in virus inactivation.

Eucalyptus essential oil can inactivate various viruses, including herpes simplex virus (Astani et al., 2010), influenza virus A (Najar et al., 2022), hepatitis A virus (Maunula et al., 2013), and coxsackievirus B3 (Elaissi et al., 2012). Moreover, EOs can interact with free viral particles (Camero et al., 2019; Catella et al., 2021; Sanna et al., 2021). This may be owing to the lipophilic properties of EOs, enabling them to destroy the lipid bilayer of the viral envelope, resulting in damage to the membrane and loss of the ability to infect cells (Madia et al., 2022). However, EOs usually have no antiviral effects against non-enveloped viruses (Cermelli et al., 2008).

In conclusion, the EEO can effectively inactivate free PRRSV particles at safe concentrations. Therefore, it would be interesting to study the antiviral effects of only the active components of EEO in the future. Furthermore, EOs are volatile and may help reduce infections caused by airborne viruses through steam evaporation; however, this requires further investigation.

## Data Availability

The raw data supporting the conclusions of this article will be made available by the authors, without undue reservation.
